# Building student loyalty in higher education: the role of corporate reputation

**DOI:** 10.12688/f1000research.129077.2

**Published:** 2023-11-30

**Authors:** Estacio Dinazarte Omar Raja

**Affiliations:** 1 Faculdade de Economia, Universidade Eduardo Mondlane, Maputo, Maputo Cidade, 1100, Mozambique

**Keywords:** Africa, corporate reputation, higher education institutions, student loyalty, Mozambique

## Abstract

**Background:**

Reputation is a vital asset for Higher Education Institutions (HEIs) as it constitutes a source of competitive advantage because it works as a quality sign for the stakeholders. Because of globalisation, decreasing funding and the changing environment in the Mozambican higher education market, HEIs need to build a favourable reputation to stay relevant. This research aims to study how a university’s reputation can influence student loyalty.

**Methods:**

Utilizing a descriptive and analytical methodology, a quantitative investigation was carried out by surveying 402 students enrolled in higher education institution (HEI) courses in Mozambique. The research employed a survey questionnaire to directly collect information from the study participants.

**Results:**

The study concluded that university reputation affects student loyalty, as the relationship between both variables is statistically significant. All dimensions of HEIs reputation, namely, academic competence, social attractiveness, and responsible management, positively impact student loyalty. Although, the latter variable did not have a statistically significant impact, contradicting the current theoretical framework.

**Conclusions:**

The study’s findings suggest that corporate reputation has a favorable impact on student loyalty, demonstrating a significant relationship between the two factors. Therefore,improve student retention, HEIs should seek to enhance their academic competence and social attractiveness.

**Furure research:**

The study suggests future research should address sample composition issues by increasing representation from underrepresented groups. It recommends a more in-depth exploration of the responsible management construct, advocating for the inclusion of context-specific indicators. Furthermore, the study proposes investigating additional factors to enhance the understanding of the relationship between university reputation and student loyalty.

## Introduction

In a globalised and increasingly competitive market characterised by increased student mobility and reduced government funding, higher education institutions (HEIs) must compete to attract the best students ( Musselin, 2018). Therefore, universities increasingly need an excellent reputation to be distinctive and relevant in the market and consequently competitive ( Valtere, 2012).

An institution’s reputation has to do with the reliability and credibility that an organisation projects to its stakeholders ( Morrissey, 2012), and reputation can be measured through the ability that this organisation has to consistently meet the expectations of its target groups over time ( Morrissey, 2012). Therefore, reputation is built over time and links to the organisation’s history ( Bickerton, 2000; Morrissey, 2012; Nguyen & LeBlanc, 2001). An HEI builds its reputation with the quality of its human resources, the provision of quality education, innovation through relevant scientific research, the quality of its infrastructure and the high degree of employability of its graduates ( Sontaite & Bakanauskas, 2011; Valtere, 2012).

It is acknowledged that corporate social responsibility, as organizations actions that considers stakeholders, impacts positively in brand reputation of higher education institutions ( Rasoolimanesh *et al.*, 2021), by influencing students’ perceptions of their reputation ( Azizi & Sassen, 2023). CSR practices in HEIs contribute to reputation through image enhancement, positive brand association, attracting like-minded students, differentiation in a competitive market, community building, alumni engagement, employability, positive media coverage, impact on rankings, and a commitment to long-term sustainability. These mechanisms collectively influence student perceptions and decisions, making CSR an integral aspect of strategic institutional management ( Rasoolimanesh *et al.*, 2021; Tan *et al.*, 2022; Azizi & Sassen, 2023).

Reputation is essential for organisations, in general, as it constitutes a strategic resource with the potential to create value ( Powell, 2016) and to obtain sustainable competitive advantage ( Grant, 2013) because it holds all properties of a strategic resource ( Grant, 2013; Hitt *et al.*, 2009; Jekins *et al.*, 2016). In the case of an HEI, reputation benefits can be the ability to attract more and better students, attract and retain the best professionals, obtain alumni support, attract funders and establish strategic partnerships with other institutions ( Suomi *et al.*, 2014). HEIs face many challenges that force them to find new sources of competitive advantage. Therefore, reputation and accreditation systems are helping universities to face new threats ( Boscor, 2015). A good reputation and image of the HEI increase student loyalty, positively affecting their intentions to collaborate with the institution in the future ( Eryilmaz, 2016). Thus, HEIs invest resources to create a favourable perception among their stakeholders ( Lafuente-Ruiz-de-Sabando *et al.*, 2017). Meanwhile, a poor reputation can undermine student loyalty by influencing enrollment decisions, affecting perceived educational value, impacting alumni relations, potentially hindering employability, and shaping the institution's overall image in the eyes of students and the broader community ( Alves & Raposo, 2010; Ismanova, 2019; Suomi, *et al.*, 2014). Thus, building and maintaining a positive reputation is crucial for fostering student loyalty and the long-term success of a higher education institution ( Eryilmaz, 2016).

The research agenda in this area of knowledge recommends that further studies be carried out in different contexts ( Abbas, 2014, 2019; Keller & Lehman, 2006; Powell, 2016), as most of the research is focused mainly on the western and Asian contexts. There are few empirical studies on this subject in the African continent. Hence, studying the Mozambican context is interesting primarily because, besides the replication of the empirical model, there is ground and opportunity to extend our knowledge about this issue by investigating what factors are set apart from the conventional theoretical models when applied to different contexts.

Besides the fulfilment of the contextual gap, this paper will contribute to the academic debate by yielding a deeper understanding of which factors are more determinant when managing the university’s reputation to improve student loyalty. Furthermore, the study aims to compare perceptions of students between private and public universities, as suggested in s previous study by Rasoolimanesh *et al.* (2021), to generalise, more confidently, the results among different types of institutions. The insights from the research can give us valuable inputs to improve and refine the model, which explains the relationship between both variables, as suggested by other authors ( Rojas-Médez *et al.*, 2009).

Similar to other countries, the landscape of the HEIs market in Mozambique has changed dramatically in the late years, with the decrease in public funding and a rapid expansion of the number of institutions and student population, resulting in an overcrowded market in the private and public sectors. This rapid transformation was seen by the public opinion with significant concerns about neglecting the educational quality, consequently hindering the image and reputation of the whole country’s tertiary education system. Additionally, in 2012, the approval of the new financing strategy for HEIs in Mozambique, which provides for the financing of higher education institutions based on performance indicators ( Ministério da Educação, 2013), comes with new challenges, especially for public institutions.

Thus, this study wishes to answer the following questions:

• Which dimensions of university reputation are determinants of student loyalty?
• Do university reputation and student loyalty differ between public and privately owned institutions?

Knowing how university reputation affects student loyalty and what factors are more critical is relevant because HEIs can focus on improving the dimensions essential for their success, depending on the context. The research will highlight that some dimensions may not be as relevant as expected.

## Literature review

Corporate reputation has been systematically and more frequently the subject of debates and discussions ( Babić-Hodović *et al.*, 2011; Eberl & Schwaiger, 2005; Petrokaite & Stravinskiene, 2013), hence the collection of scientific publications on the subject ( Maden *et al.*, 2012; Shamma, 2012). The new dynamics in the relationship between the organisation and its interest groups have increasingly highlighted reputational management as the relevant and determining element ( Petrokaite & Stravinskiene, 2013). Proper corporate reputation management can be critical for the success of HEIs in a highly competitive environment ( Del-Castillo-Feito *et al.*, 2019; Munisamy *et al.*, 2014).

### Corporate reputation

Reputation is a multidisciplinary concept, so there are several definitions with different perspectives in the literature ( Eberl & Schwaiger, 2005; Maden *et al.*, 2012). Several branches of knowledge, such as economics, marketing, management, psychology and sociology, have studied the concept ( Ponzi *et al.*, 2011). Schwaiger (2004) argues that there are different angles to view corporate reputation: corporate brand, representation of the firm’s goodwill, corporate identity, the barrier to market entry for potential competitors, and a signal of the organisation’s actions and future behaviour. Therefore, the concept is multifaceted and incorporates interrelated characteristics such as credibility, reliability and responsibility ( Carmeli & Cohen, 2001; Morrissey, 2012; Puncheva-Michelotti & Michelotti, 2010).

Nguyen and LeBlanc (2001) consider credibility as part of corporate reputation, which may result from the contrast between its promises and actions. Corporate reputation is built over time, resulting from accumulated judgements and opinions from different stakeholders ( Nguyen & LeBlanc, 2001).

Reputation in an HEI can be defined simply as ‘the collective representation maintained over time by the multiple constituents (internal and external) of a university’ ( Alessandri *et al.*, 2006). The definition of reputation as presented by Alessandri *et al.* (2006) provides a holistic and dynamic framework that aligns well with the central theme of the article, which is the role of corporate reputation in building student loyalty in higher education. It captures the multifaceted nature of reputation and its impact on various constituents over time. Sontaite and Bakanauskas (2011) argue that corporate reputation results from a subjective and collective assessment of the institution by the stakeholders based on its performance, potential, brand value, communication and past behaviour. Also, in line with the same authors, they claim that managing a university’s reputation is challenging when its constituent units, such as faculties and schools, have a distinct reputation among stakeholders. On the other hand, the institution may have a reputation locally, while internationally, it does not have the same recognition ( Suomi *et al.*, 2014).

A definition that summarises the various perspectives presented on the concept describes corporate reputation as ‘the collective representation of the past and present actions and results of the organisation, which describes its ability to obtain different results from different stakeholders’ ( Martin de Castro *et al.*, 2006).

*Sources of corporate reputation*

Shamma (2012) presents a list of possible sources of corporate reputation: the communication and behaviour of workers, people’s experiences with the organisation, the organisation’s communications, the media’s interpretation of the organisation, word-of-mouth, competition and rumours.

This concept is formed based on interactions and transactions of stakeholders with the organisation ( Eberl & Schwaiger, 2005). Reputation also results from the organisation’s activity in the past, but with an impact on the present behaviour of stakeholders concerning the organisation ( Petrokaite & Stravinskiene, 2013). For this reason, reputation takes years to build, which is why it is one of the most challenging resources to accumulate for any organisation ( Martin de Castro *et al.*, 2006). However, it is fragile because any action that harms a specific group can jeopardise the accumulated organisation’s prestige or credibility over time ( Nguyen & LeBlanc, 2001).

Corporate reputation links to stakeholder theory ( Feldman *et al.*, 2014) so the perception of corporate reputation varies depending on the stakeholder group ( Petrokaite & Stravinskiene, 2013). According to Helm (2007), the different groups of stakeholders are customers, investors, workers, suppliers and society. The student is an essential stakeholder of an HEI, and his family has an important role, as it influences his choices and preferences ( Suomi, 2014).

*Corporate reputation measurement*

Corporate reputation is a difficult concept to measure, and there is no consensus between academics and practitioners on the subject ( Sontaite & Bakanauskas, 2011). Possession of characteristics such as causal ambiguity, social complexity, and slow accumulation associated with the organisational history makes this concept have a high level of intangibility which is why it is a complex construct to measure ( Martin de Castro *et al.*, 2006). However, Sontaite and Bakanauskas (2011) sustain that the indicators related to the creation of good or bad attitudes of stakeholders concerning the organisation are the basis for reputation measurement, and such indicators must be weighted differently depending on the importance of each stakeholder.

Caruana and Chircop (2000) present four components that constitute the reputation construct: affection or emotional appeal, image, level of awareness about the organisation, the result of its behaviour and past performance, and the experiences and interactions of stakeholders with the organisation. For Helm (2007), quality of products and services, emotional appeal, vision and leadership, results of financial activity, social responsibility and working conditions constitute the dimensions of corporate reputation.

Telci and Kantur (2014) developed and validated a scale to measure the reputation of universities. The scale consists of 20 indicators divided into three dimensions: academic competence, social attractiveness and responsible management. The academic competence construct reflects the general educational capacity or quality of the university and also the quality of services provided by the institution. In comparison, social attractiveness refers to items such as the innovative capacity of the university among side its physical conditions. Finally, the dimension of responsible management regards the responsibilities of the university management to its stakeholders ( Telci & Kantur, 2014). The present study adopted this scale to measure the reputation of HEIs because of the context similarity. The methodological procedure suggested to measure the reputation of HEIs is the use of questionnaires applied to stakeholders.

### Student loyalty

Student loyalty is the student’s intention to recommend some type of relationship with the institution to third parties, as well as their satisfaction with the choice of the institution or course they attend and the predisposition to relate to the institution in the future ( Østergaard & Kristensen, 2005). The student’s tendency to choose the same institution over another to satisfy a particular need is also related to this concept ( Temizer & Turkyilmaz, 2012).

Student loyalty is affected by a good university image and student satisfaction ( Temizer & Turkyilmaz, 2012). Student retention also depends on other factors such as student’s academic abilities (Pantages & Creedon, 1978 *in* Kara & Deshields, 2004), their adjustment and social integration into the educational institution (Gerdes & Mallinckrodt, 1994; Mallinckrodt, 1988 *in* Kara & Deshields, 2004) and the expectations formed before enrolling at the institution (Baker, McNeil and Sirky, 1985 *in* Kara & Deshields, 2004). It is important to note that the student’s motivation to obtain an academic degree and commitment to the institution are also important factors for student retention ( Kara & Deshields, 2004).

Student loyalty plays a crucial role in the success of HEIs, as loyalty not only perpetuates the institutions’ potential revenues but also causes positive synergies through a positive recommendation about the institution ( Shahsavar & Sudzina, 2017). The increase in the student retention rate positively affects the life cycle of tuition fees, and it generates synergies in the low-cost recruitment of new students through word-of-mouth recommendations ( Kara & Deshields, 2004). Additionally, the probability of retaining a student at a university increases if he/she stays for an extended period in that institution because of the high transaction costs for the eventual move to a competing institution ( Kara & Deshields, 2004).

*Student loyalty measurement*

Student loyalty has attached to its measurement the following indicators: the intention to choose the same university, the recommendation to others and the intention to leave the university when possible ( Temizer & Turkyilmaz, 2012). Blackmore *et al.* (2006) in Douglas, Mcclelland, & Davies (2008) present the following indicators of student loyalty: the student’s predisposition to continue their studies at the institution, the level and frequency of use of the university’s services and the intention of recommending the institution or course to friends, neighbours or family members translate the concept of student loyalty.

Taecharungroj (2014) uses a scale that groups these three categories into five items, namely: saying positive things about the university to other people; intention to recommend the university to anyone seeking advice; encouraging friends and family to consider the university and to have a close relationship with it; consider the institution as the first choice when choosing a university to study, and the intention of relating to the university in the future. The present research adopts this last scale to measure student loyalty.

### Relationship between corporate reputation and student loyalty

A favourable corporate image and reputation influence the attitudes of the organisation’s stakeholders ( Iwu-Egwuonwu, 2011). Among other antecedents, such as student satisfaction, the reputation of the university is one of the key drivers of student loyalty ( Alves & Raposo, 2010; Helgesen & Nesset, 2007; Kaushal & Ali, 2020; Nesset & Helgesen, 2009) Because of these arguments the study proposes to test the following hypothesis:

**H1:** Corporate reputation has a significant relationship with student loyalty.

University reputation for this research contains three main dimensions: academic competence, social attractiveness and responsible management ( Telci & Kantur, 2014). For this reason, theoretically, it is plausible to establish the link between these dimensions and student loyalty.

Prior studies have shown that the quality of education an institution provides ( Daud *et al.*, 2020; Ismanova, 2019; Lin & Tsai, 2008; Nesset & Helgesen, 2009; Yang *et al.*, 2008), the trustworthiness of the university ( Carvalho & de Oliveira Mota, 2010; Ismanova, 2019; Rojas-Méndez *et al.*, 2009), a commitment to academic excellence ( Vianden & Barlow, 2014), and graduates’ success ( Quintal & Phau, 2016) are crucial ingredients of student loyalty towards the university. Those factors are all items of academic competence in an HEI ( Telci & Kantur, 2014). Thus, the following hypothesis arises:

**H2**: Academic competence has a significant relationship with student loyalty.

The quality of the facilities ( Nesset & Helgesen, 2009; Thomas, 2011; Vianden & Barlow, 2014) such as having a good campus, quality of libraries, among others, and the overall experience the student ( Yu & Kim, 2008) has in an HEI affects student loyalty positively. That’sThat’sThat’s why the more social attractive an institution is, the more loyalty it gets from its students ( Telci & Kantur, 2014). This argument suggests the following hypothesis:

**H3:** Social attractiveness has a significant relationship with student loyalty.

The reputation of the institution’s top management is one of the critical elements of HEIs reputation management. This factor helps to establish good relationships with both internal and external stakeholders, reinforcing their confidence in the university’s administration ( Zyryanova *et al.*, 2020), which in turn has a positive impact on student loyalty ( Carvalho & de Oliveira Mota, 2010; Telci & Kantur, 2014). Thus, the author formulates the following hypothesis:

**H4:** Responsible management has a significant relationship with student loyalty.

Strategically, public HEIs increasingly mirror private sector practices ( Buckland, 2009). However, Khan *et al.* (2018) have compared the management practices of private and public universities and found that, although both use similar managerial practices, there are differences in some aspects, such as leadership style and management experience. This view about the existence of different settings between public and private institutions may create different perceptions about their reputations and, consequently, relationships with their stakeholders. For this reason, it is reasonable using the type of institution, whether public or private, as the control variable on the behaviour of both the dependent and independent variables.

Student perceptions and assessments about their HEI may vary across public and private institutions ( Mahmoud & Grigoriou, 2017) due to switching costs ( Maden *et al.*, 2012), cost of education ( Goh *et al.*, 2017), perceived value ( Goh *et al.*, 2017; Taecharungroj, 2014) and overall student experience ( Yu & Kim, 2008). Because this study defines university reputation and student loyalty based on student perceptions and evaluations of their institutions, therefore, it is reasonable to expect that:

**H5:** There is a significant difference in the level of corporate reputation between public and private HEIs.

**H6:** There is a significant difference in student loyalty between public and private HEIs.

**Figure 1.**
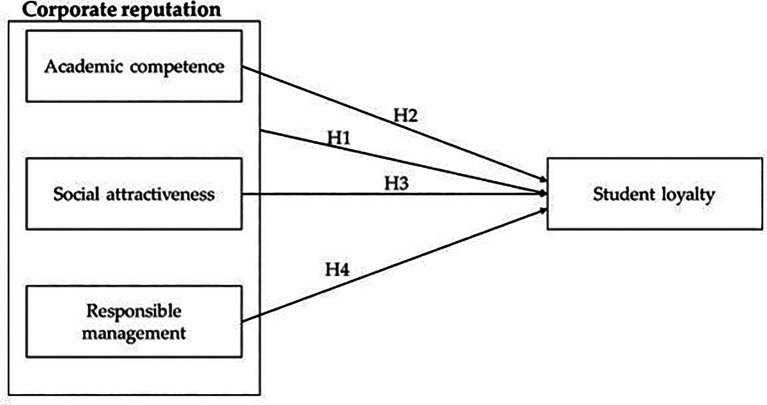
Conceptual model of the study.

See Figure 1 for the conceptual model of the study.

## Methods

The purpose of this study is to find out and explain the relationship between university reputations of Mozambican public and private institutions and student loyalty. The research design of the study is descriptive and analytical. The research is based on a quantitative survey and analysis. The main instrument for data collection was a closed-question questionnaire consisting of items from the scales of the primary constructs of the study, namely corporate reputation and student loyalty. The items on the scales are measured by a five-point Likert scale, which has the following description, strongly disagree, tend to disagree, neutral, tend to agree, and strongly agree. Respondents provided written informed consent to fill the questionnaire voluntarily, and anonymity and confidentiality were granted to comply with ethical issues. A scientific board approved the ethical compliance of the study, and the author presented the project for approval before a jury, in 2019, as part of his PhD thesis.

The Scientific Commission of The Faculty of Economics of UEM approved the ethical compliance of the study (Approval number: 000350/FACECO/2023).

The unit of analysis of this study is public and private higher education institutions operating in Mozambique. The sample unit is students currently attending a course at a higher education institution in the country. The sampling technique was convenience and snowball, and the study’s sample size was 384 respondents. However, 402 questionnaires were collected with 396 valid responses.

The data was collected online through an electronic survey platform from August to October 2021, where students attending different HEIs from all the regions of Mozambique filled out the questionnaire. The researcher reached out to universities, professors, and university students, providing a link to individuals who met the sample criteria, asking them to fill out the questionnaire and distribute it to others with similar profiles. As the author is a university faculty member, the snowball sampling method was combined with convenience sampling by also surveying students from the author’s institution.

For data analysis and testing, SPSS version 20 was used. The collected data were subjected to a series of statistical tests, such as multivariate regression, bivariate analysis, ANOVA test, and exploratory factor analysis.

## Results

### Social characterisation and academic profile of respondents

The sample has a slightly higher male representation (50.5%), with the most expressive age group being 21 years old or less with 41%, slightly above the 22-30 age group, representing 40.8%. Regarding educational establishments, 82% of respondents belong to public institutions. Universities represent 88.4%, followed by Higher Institutes with 10.4%. The first three years concentrate a higher proportion of students (62.8%). See Table 1.

**Table 1.** Social characterization and academic profile of respondents.

Variable		N	%	Variable		N	%
Age	up to 21 years	162	41.0%	Year of attendance	1st year	81	20.5%
	22-30 years	161	40.8%		2nd year	82	20.8%
	>30 years	72	18.2%		3rd year	85	21.5%
	Total	395	100%		4th year	103	26.1%
Sex	Female	199	50.5%		5th year	36	9.1%
	Male	195	49.5%		6th year	8	2.0%
	Total	394	100%		Total	395	100%
				Level	Graduation	350	89.3%
					Master's degree	38	9.7%
					Doctorate	4	1.0%
					Total	392	100%
				Nature of the institution	Public	328	82.8%
					Private	68	17.2%
					Total	396	100%
				Type of institution	University	350	88.4%
					Superior Institute	41	10.4%
					College	4	1.0%
					Academy	1	0.3%
					Total	396	100%

### Reliability of constructs

The original authors tested already the indicators of the scales of the main constructs of the present study and presented a high index of reliability measured through Cronbach’s Alpha. However, to reinforce the validity of the scales in the Mozambican context, the author tested the reliability of the constructs, and the results showed that all constructs have good reliability. See Table 2.

**Table 2.** Composition and reliability of synthetic indexes.

Synthetic index	Mean	*Std. Deviation*	*Cronbach's Alpha*	Nr. of Indicators
Corporate reputation	3.72	0.63562	0.92	20
Student loyalty	3.67	1.00695	0.87	5
Academic competence	3.91	0.67882	0.89	11
Social attractiveness	3.34	0.80338	0.81	6
Responsible management	3.72	0.74929	0.71	3

### Hypotheses testing

**H1:** Corporate reputation has a significant relationship with student loyalty.

The results show that the independent variables (academic ability, social attractiveness and responsible management) explain in 45% (adjusted R ^2^) the variation of the dependent variable (Student Loyalty). Therefore, the model manages to explain almost half of the dependent variable, leaving a significant part of 55% explained by other variables. The hypothesis of R ^2^ being zero being statistically significant (F(3.391)=109,627; p<0.001) is rejected.

The independent variables (Academic competence, social attractiveness and responsible management) show a statistically significant correlation (p<0.00) with the independent variable (Student loyalty). Correlations range from moderate for Responsible management (0.442) and Social attractiveness (0.588) to high for Academic competence (0.63). The coefficient table shows the equation of the estimated line which is: Student Loyalty=-0.187+0.622 Academic competence+0.388 Social attractiveness+0.032 Responsible management.

**H2:** Academic competence has a significant relationship with student loyalty.

**H3:** Social attractiveness has a significant relationship with student loyalty.

Academic competence (p<0.001) and social attractiveness (p<0.001) have both a high significance in the dependent variable. The results confirmed the hypotheses positively.

**H4:** Responsible management has a significant relationship with student loyalty

The variable responsible management, in addition to having a shallow impact on the dependent variable (H4), that is, in the model, not an explanatory determinant of student loyalty, and this relationship is not statistically significant (p=0.629). Thus, H4 was not verified.

**H5:** There is a significant difference in the level of corporate reputation between public and private educational institutions.

In public (3.71) and private (3.74) institutions, corporate reputation is higher than the centre of the scale, slightly higher in private institutions. The difference in means is not statistically significant (H5) (t(393) =-0.327; p=0.326). Students from private institutions believe their institutions have a slightly higher reputation. A slightly higher percentage (38.2%) believe that the reputation level of their institutions is high. In comparison, only 37% of students from public higher education institutions say the same. The hypothesis H5 was not validated.

**H6:** There is a significant difference in the level of student loyalty between public and private educational institutions.

As for the level of student loyalty, the results show that in both public and private institutions, student loyalty is higher at the centre of the scale, being slightly higher in public institutions. The difference in means is statistically significant (H6) (t(87793)=2,373; p=0.04). Students from public institutions showed more loyalty towards their institutions. A higher percentage (48%) of students from public institutions claimed to be very loyal. In contrast, the percentage of students from private institutions said the same was 36.8%. The hypothesis was confirmed ( Table 3).

**Table 3.** Summary of hypothesis.

Hypothesis	Result	P value
H1: Corporate reputation has a significant relationship with student loyalty.	Confirmed	0.000
H2: Academic competence has a significant relationship with student loyalty.	Confirmed	0.000
H3: Social attractiveness has a significant relationship with student loyalty.	Confirmed	0.000
H4. Responsible management has a significant relationship with student loyalty.	Not supported	0.629
H5: There is a significant difference in the level of corporate reputation between public and private educational institutions.	Not supported	0.326
H6: There is a significant difference in the level of student loyalty between public and private educational institutions.	Confirmed	0.04

## Discussion

This study aimed to investigate the influence of university reputation and its dimensions, namely academic competence, social attractiveness and responsible management, on student loyalty. The research targeted student loyalty as a dependent variable as previous studies proved that loyalty plays a crucial role in creating the success of HEIs, which is why it requires special attention and resources. This variable impacts increasing the funding of institutions through high retention rates and lasting relationships and synergies through positive recommendations, by students and other stakeholders, about the institution ( Kara & Deshields, 2004; Shahsavar & Sudzina, 2017).

The study’s empirical evidence showed a positive and significant relationship between university reputation and student loyalty in the context of HEIs in Mozambique, both for public and private institutions. The results of the study confirmed previous thoughts that the reputation of an HEI helps to increase student loyalty, positively affecting their intentions to collaborate with the institution in the future ( Eryilmaz, 2016). Thus, reinforcing the belief that corporate reputation is a strategic asset crucial for value creation or the production of tangible results ( Powell, 2016), allowing it to create conditions for obtaining sustainable competitive advantage ( Grant, 2013). For this reason, HEIs invest a significant part of their resources in having a favourable perception among their stakeholders, emphasising the students ( Lafuente-Ruiz-de-Sabando *et al.*, 2017).

The study also confirmed previous knowledge that academic competence and social attractiveness are crucial in influencing student loyalty, as the regression analysis demonstrated a significant relationship between those dimensions and the dependent variable. When students perceive that the HEIs provide them with quality education and a robust physical and social infrastructure, they tend to be more loyal to the institution ( Daud *et al.*, 2020; Ismanova, 2019; Lin & Tsai, 2008; Nesset & Helgesen, 2009; Vianden & Barlow, 2014; Yang *et al.*, 2008).

The research could not confirm the significant impact of the variable responsible management on student loyalty. At the same time, theory suggests that a favourable top management reputation is critical to building solid relationships with the university stakeholders (( Zyryanova *et al.*, 2020). Strong leadership and good governance are vital components of corporate reputation ( Suomi, 2014). Thus, it is wise not to completely rule out the importance of trust in university good administration practices from the model.

The study also aimed to compare students’ perceptions of reputation and loyalty between public and private HEIs to assess if there are significant differences that would justify different treatment of each group of institutions. The results showed that while the differences in university reputation between the two groups are not significant, there are differences in student loyalty between public and private HEIs. Students from public HEIs showed higher loyalty compared with private institutions. What stands out about the results is that besides recognising the effect of university reputation on student loyalty, other factors influence the behaviour of the dependent variable.

## Conclusions, implications and limitations

### Conclusions

The study concluded that corporate reputation positively affects student loyalty. Corporate reputation can explain 45% (adjusted R ^2^) of the variance of the dependent variable, student loyalty. The relationship between the two variables is significant. The dimensions of corporate reputation with the most impact are academic competence and social attractiveness. The results showed that responsible management has a residual and non-significant impact on student loyalty.

The results also allow us to conclude that both corporate reputation and student loyalty have a moderate level and above the average of the scale in the respondents’ statements, both for public and private institutions. As these variables are positively correlated, it is expected that they behave accordingly.

The analyses show no significant differences between public and private institutions regarding institutional reputation. However, as for student loyalty, the results attest to a statistically significant difference between the nature of the institutions, pointing to a greater intensity of loyalty in public educational institutions.

### Theoretical and managerial implications

This study contributes to the literature by providing empirical evidence of a different context in which university reputations play a key role in fostering student loyalty, both from private and public HEIs. The research results showed that strengthening the activities of the HEIs’ core business, which is the improvement of their academic competence, proves to be a logical path to the success of the institutions, as this dimension is the one that reveals the most significant impact on student loyalty.

The study’s practical implications are that it gives a framework for HEIs administrators, both from public and private institutions, to manage the factors of university reputation that better affect student loyalty. Given that academic competence and social attractiveness were identified as the dimensions of corporate reputation with the most impact on student loyalty, Mozambican HEIs should prioritize efforts to enhance these aspects. Thus, to retain their students, Mozambican HEIs must focus on continuous improvement of academic programs, faculty quality, and educational resources to strengthen academic competence. They must also Invest in creating a vibrant and inclusive campus culture, fostering social interactions, and providing essential resources to enhance social attractiveness. These actions will significantly impact student loyalty, which is a fundamental element of the success of higher education institutions in an increasingly competitive environment. Specifically, Mozambican HEIs managers have empirical evidence from scientific research that could back up their decisions to allocate resources to improve their reputation and ultimately, affecting, positively student loyalty.

The lack of significance for responsible management might indicate that the specific cultural, economic, or social context in Mozambique places less emphasis on this dimension when students evaluate their loyalty to an institution. However, institutions should not completely disregard it. It may be an area where improvements could lead to a more positive impact on student loyalty. Continuous monitoring, feedback, and adjustments in responsible management practices could be explored to assess their potential influence on student loyalty over time.

### Limitations and direction for future research

The present study has some limitations that are worth considering. First, the sample composition can be improved as, for example, there is a lower representation of respondents from private sector institutions with 17.2%, while the representation in the population is 39.8%. The other underrepresented groups are the post-graduate students (10.7%) and students enrolled in Academies, Colleges and Superior Institutes (11.7%). Thus, for future research, to generalise the results with more confidence, it is recommended to increase the proportion of representatives of those groups, as it might yield richer insights.

Secondly, bearing in mind that the responsible management construct was the one that presented the lowest degree of reliability of Cronbach’s Alpha (0.71), it is recommended that for future research, a deeper exploration of the construct with the inclusion of other indicators that reflect and adapt to the local context. Previous research ( Zyryanova *et al.*, 2020) pointed out that trust in managerial capabilities and responsibilities is crucial to engaging relationships with key stakeholders. Thus, a better understanding of the constructs and how they fit into the model is worth considering. Future research could also delve deeper into understanding why responsible management does not significantly impact student loyalty in the Mozambican context. This could involve qualitative research methods, interviews, or surveys exploring students' perceptions and attitudes towards responsible management practices.

Finally, the model can only explain 45% (adjusted R ^2^) of the variance of the dependent variable. The results showed no significant differences in reputation between public and private universities. While regarding student loyalty, the result presented significant differences between both groups. This finding may suggest that there are other variables intervening in the model. Thus, to better understand the mechanisms of the relationship between university reputation on student loyalty, other variables such as service quality ( Teeroovengadum *et al.*, 2019), student satisfaction ( Alves & Raposo, 2006), image ( Alves & Raposo, 2006; Quintal & Phau, 2016; Teeroovengadum *et al.*, 2019), trust ( Bergamo *et al.*, 2012; Bowden, 2011; Goh *et al.*, 2017; Ismanova, 2019; Rasoolimanesh *et al.*, 2021), switching costs ( Maden *et al.*, 2012) and student commitment ( Ismanova, 2019; Rojas-Médez *et al.*, 2009; Snijders *et al.*, 2020), should be included in the model to explain the variance on the dependent variable. Further studies may include mediators in the model or even change the role of university reputation in the model to explain its relationship with student loyalty.

## Data Availability

Figshare: Survey_of_Moz_HEis_Students_clean.csv, https://doi.org/10.6084/m9.figshare.20212751.v1 ( Raja, 2022). Figshare: Questionnaire reputation and student loyalty.pdf, https://doi.org/10.6084/m9.figshare.23844462.v1 ( Raja, 2023a). Figshare: Appendix_1.docx (Items of the university reputation scale), https://doi.org/10.6084/m9.figshare.23843316.v1 ( Raja, 2023b) Data are available under the terms of the Creative Commons Attribution 4.0 International license (CC-BY 4.0)
