# Supplementary material for: Building student loyalty in higher education: the role of corporate reputation
Source: F1000Res. 2023 Dec 18;12:1102. Originally published 2023 Sep 4. [Version 3] doi: 10.12688/f1000research.129077.3 (PMC10716606; doi:10.12688/f1000research.129077.3)
Supplement: Peer review history

# Reviewer report: Reviewer response for version 2

**DOI:** 10.5256/f1000research.159393.r227204

**Published:** 2023-12-12

**Authors:** Poh Ling Tan

**Affiliations:** 1 Department of Advertising, Xiamen University Malaysia, Sepang, Malaysia

I accept all changes.

Is the work clearly and accurately presented and does it cite the current literature?

Yes

If applicable, is the statistical analysis and its interpretation appropriate?

Yes

Are all the source data underlying the results available to ensure full reproducibility?

Yes

Is the study design appropriate and is the work technically sound?

Yes

Are the conclusions drawn adequately supported by the results?

Partly

Are sufficient details of methods and analysis provided to allow replication by others?

Partly

Reviewer Expertise:

Communication Strategies / Branding / CSR

I confirm that I have read this submission and believe that I have an appropriate level of expertise to confirm that it is of an acceptable scientific standard.

# Reviewer report: Reviewer response for version 2

**DOI:** 10.5256/f1000research.159393.r227205

**Published:** 2023-12-12

**Authors:** Pilar Zorrilla

**Affiliations:** 1 Department of Marketing, University of the Basque Country, Leioa, Spain

The reference cited with an error has not been corrected. The correct referece is: 

 Lafuente-Ruiz-de-Sabando A, Zorrilla P, **Forcada, J.** : A Review of Higher Education Image and Reputation Literature: Knowledge Gaps and Research Agenda. *Eur. Res. Manag. Bus. Econ.* 2017; **282**: 1043–1064.

 About the other considerations, I see have been resolved

Is the work clearly and accurately presented and does it cite the current literature?

Yes

If applicable, is the statistical analysis and its interpretation appropriate?

Partly

Are all the source data underlying the results available to ensure full reproducibility?

Yes

Is the study design appropriate and is the work technically sound?

Yes

Are the conclusions drawn adequately supported by the results?

Yes

Are sufficient details of methods and analysis provided to allow replication by others?

Yes

Reviewer Expertise:

Marketing

I confirm that I have read this submission and believe that I have an appropriate level of expertise to confirm that it is of an acceptable scientific standard.

# Reviewer report: Reviewer response for version 2

**DOI:** 10.5256/f1000research.159393.r227206

**Published:** 2023-12-12

**Authors:** Sallaudin Hassan

**Affiliations:** 1 Malaysian Institute of Industrial Technology (MITEC), Universiti Kuala Lumpur, Kuala Lumpur, Federal Territory of Kuala Lumpur, Malaysia

Based on my review, it is found that the author already corrected and improved the writing. 

 Recommended for next process.

Is the work clearly and accurately presented and does it cite the current literature?

Yes

If applicable, is the statistical analysis and its interpretation appropriate?

Yes

Are all the source data underlying the results available to ensure full reproducibility?

Yes

Is the study design appropriate and is the work technically sound?

Yes

Are the conclusions drawn adequately supported by the results?

Yes

Are sufficient details of methods and analysis provided to allow replication by others?

Yes

Reviewer Expertise:

Quality Management System, Operation Management, Supply Chain Management, Service Quality, TVET Education studies.

I confirm that I have read this submission and believe that I have an appropriate level of expertise to confirm that it is of an acceptable scientific standard.

# Reviewer report: Reviewer response for version 1

**DOI:** 10.5256/f1000research.141734.r214486

**Published:** 2023-11-06

**Authors:** Poh Ling Tan

**Affiliations:** 1 Department of Advertising, Xiamen University Malaysia, Sepang, Malaysia

The introduction jumps quickly into the topic without establishing the broader relevance of corporate social responsibility (CSR) in the context of HEIs. It could benefit from a sentence or two explaining how CSR specifically ties into the reputation and competitive advantage of HEIs.

 The introduction could benefit from a clearer explanation of how Corporate Social Responsibility (CSR) specifically influences the reputation of higher education institutions. While the introduction discusses reputation at length, the direct impact of CSR initiatives on reputation and student loyalty is not explicitly stated. The author should consider detailing the mechanisms through which CSR practices contribute to the reputation of HEIs, potentially influencing student perceptions and decisions, and how this might differ in the context of Mozambique compared to other regions.

 While the study highlights the significant impact of academic competence and social attractiveness on student loyalty, it would be beneficial to discuss the implications of responsible management's non-significant impact. The author could speculate on why this might be the case and what it could mean for HEIs' CSR strategies.

 The author notes that both corporate reputation and student loyalty are above average, but does not elaborate on the implications of this finding. It would be insightful to discuss whether this is consistent with expectations and how it compares to other studies in similar contexts.

 Address the Unique Context of Mozambique: The recommendations are somewhat generic. The author could provide more tailored advice for Mozambican HEIs, considering the country's specific educational, economic, and cultural context.

Is the work clearly and accurately presented and does it cite the current literature?

Yes

If applicable, is the statistical analysis and its interpretation appropriate?

Yes

Are all the source data underlying the results available to ensure full reproducibility?

Yes

Is the study design appropriate and is the work technically sound?

Yes

Are the conclusions drawn adequately supported by the results?

Partly

Are sufficient details of methods and analysis provided to allow replication by others?

Partly

Reviewer Expertise:

Communication Strategies / Branding / CSR

I confirm that I have read this submission and believe that I have an appropriate level of expertise to confirm that it is of an acceptable scientific standard, however I have significant reservations, as outlined above.

# Reviewer report: Reviewer response for version 1

**DOI:** 10.5256/f1000research.141734.r214479

**Published:** 2023-10-24

**Authors:** Pilar Zorrilla

**Affiliations:** 1 Department of Marketing, University of the Basque Country, Leioa, Spain

Starting from the consideration the relevance of the topic discussed, namely, the impact of reputation on student loyalty in the framework of Higher Education. This is a topic of interest that is addressed in this specific case in a geographical context, Mozambique, where there does not seem to be any research on the matter. Therefore, one of the essential contributions of the work is just this: covering the research gap in Africa context.

 The research questions are relevant and well focused. 

- Which dimensions of university reputation are determinants of student loyalty?
- Do university reputation and student loyalty differ between public and privately owned institutions?

Besides, the answer to these questions allows us to address the implications for reputation management in universities.

 The strategic importance of image and reputation of organisations is widely recognized. However, despite the extensive body of knowledge generated around the concept of reputation in organisations, researchers continue to underscore the existence of important conceptual inconsistencies surrounding them. In this paper, the author recognizes that reputation is a multidisciplinary concept but assumes a definition as a valid definition. The lack of conceptual clarity is one of the most significant research gap, so I would like to read a better argumentation about why the author has chosen Alessandri et al as the best definition of reputation.

 The following quote contains errors in one of names:  Lafuente-Ruiz-de-Sabando A, Zorrilla P, **Forcada, J**: A Review of Higher Education Image and Reputation Literature: Knowledge Gaps and Research Agenda. Eur. Res. Manag. Bus. Econ. 2017; **282**: 1043–1064.

 There are problems in several of the links to the articles cited in the bibliography.

Is the work clearly and accurately presented and does it cite the current literature?

Yes

If applicable, is the statistical analysis and its interpretation appropriate?

Partly

Are all the source data underlying the results available to ensure full reproducibility?

Yes

Is the study design appropriate and is the work technically sound?

Yes

Are the conclusions drawn adequately supported by the results?

Yes

Are sufficient details of methods and analysis provided to allow replication by others?

Yes

Reviewer Expertise:

Marketing

I confirm that I have read this submission and believe that I have an appropriate level of expertise to confirm that it is of an acceptable scientific standard, however I have significant reservations, as outlined above.

# Reviewer report: Reviewer response for version 1

**DOI:** 10.5256/f1000research.141734.r214490

**Published:** 2023-10-24

**Authors:** Sallaudin Hassan

**Affiliations:** 1 Malaysian Institute of Industrial Technology (MITEC), Universiti Kuala Lumpur, Kuala Lumpur, Federal Territory of Kuala Lumpur, Malaysia

This paper is related to the current issue especially in the effort to increase number of student and keep them retain on the same university. 

 Perhaps, please consider the following input for improvement: 

1. Abstract- please include briefly on future research. 
2. Introduction - please clearly indicate the impact of poor reputation toward student loyalty.
3. Literature review - please consider to more reference for higher TVET institution as well.  Prior to the developing of hypothesis, please clearly relate this study with related underpinning theory. In addition, need to ensure that the hypothesis is developed based on past research. 
4. Method - sampling method needs to clearly defined. 
5. Result- need to clearly explain the current performance.
6. Discussion - adequate. However, better to have supporting docs. 

Is the work clearly and accurately presented and does it cite the current literature?

Yes

If applicable, is the statistical analysis and its interpretation appropriate?

Yes

Are all the source data underlying the results available to ensure full reproducibility?

Yes

Is the study design appropriate and is the work technically sound?

Yes

Are the conclusions drawn adequately supported by the results?

Yes

Are sufficient details of methods and analysis provided to allow replication by others?

Yes

Reviewer Expertise:

Quality Management System, Operation Management, Supply Chain Management, Service Quality, TVET Education studies.

I confirm that I have read this submission and believe that I have an appropriate level of expertise to confirm that it is of an acceptable scientific standard, however I have significant reservations, as outlined above.
